
JOURNAL INFORMATION
==============================
NLM Title Abbreviation: Wirtschaftsdienst
Journal ID: Wirtschaftsdienst
NLM Journal ID: 101086612

Wirtschaftsdienst (Hamburg, Germany : 1949)

ISSN: 0043-6275

ARTICLE INFORMATION
==============================
PMCID: PMC7896164
PMID: 33642644
DOI: 10.1007/s10273-021-2847-z
Article ID: 2847
Article version: 1
Subjects: Zeitgespräch

Der Fonds für Erholung und Resilienz: eine wirtschaftliche Analyse

Gros Daniel danielg@ceps.be

grid.22793.3d 0000 0004 0609 4239 Centre for European Policy Studies (CEPS), 1 Place du Congrès, 1000 Brussels, Belgien
Electronic publication date: 2021 Feb 20
Print publication date: 2021
Volume: 101
Issue: 2
First page: 87
Last page: 90
Copyright: © Der/die Autor:in(nen) 2021
License: Open Access: Dieser Artikel wird unter der Creative Commons Namensnennung 4.0 International Lizenz veröffentlicht (http://creativecommons.org/licenses/by/4.0/deed.de).
Open Access wird durch die ZBW–Leibniz-Informationszentrum Wirtschaft gefördert.
License URL: https://creativecommons.org/licenses/by/4.0/

issue-copyright-statement: © The Author(s) 2021

==============================

Die Aufbau- und Resilienzfazilität (RRF – Recovery and Resilience Facility) stellt einen wichtigen Schritt auf dem Weg zur europäischen Solidarität dar und bietet eine neue Quelle für sichere europäische Anleihen. Obwohl sich ihre Umsetzung wahrscheinlich verzögern wird, hat schon die Ankündigung starke Auswirkungen auf die Finanzmärkte gehabt, mit niedrigeren Risikoprämien für

**Dr. Daniel Gros** ist Direktor des Centre for European Policy Studies (CEPS) in Brüssel, Belgien.

wichtige Länder wie Italien oder Spanien und einer stärkeren Nachfrage nach Euro-Vermögenswerten aus der übrigen Welt. Denn die Europäische Union hat gezeigt, dass es einen Mechanismus gibt, um bedürftigen Ländern zu helfen.

An dieser Stelle möchte ich eine kurze wirtschaftliche Bewertung der RRF vornehmen, die auf grundsätzlichen Überlegungen beruht. Jede wirtschaftliche Analyse der RRF muss von einem einfachen, aber grundlegenden Satz ausgehen: „Geld ist fungibel". Dies gilt in mehreren Dimensionen.

## Geld ist im Laufe der Zeit fungibel

Der größte Teil der Mittel aus der RRF wird erst nach 2022 ausgezahlt werden, d. h. nachdem die Krise (hoffentlich) überwunden worden ist. Es wäre jedoch ein Fehler, aus dieser verzögerten Auszahlung zu schließen, dass die RRF während der Krise irrelevant sei. Zunächst hat schon die Ankündigung der im Juli 2020 erzielten Vereinbarung des Europäischen Rates die Märkte stabilisiert und den Marktzugang auch für Länder mit hohen Schulden gesichert. Bereits im März und April 2020 hatte die EZB durch energisches Eingreifen das Entstehen einer Liquiditäts- und Finanzkrise verhindert. Einige Risikospreads waren jedoch relativ hoch geblieben.

Nach der Einigung über die RRF verbesserte sich der Marktzugang weiter, und die Kreditkosten für die Peripherieländer gingen zurück. Der italienische Staat kann jetzt mit Zehn-Jahres-Zinssätzen um 1 % rechnen, fast ein Allzeittief, weniger als noch kurz vor der Krise, im Januar 2020. Die Renditen der spanischen zehnjährigen Anleihen liegen jetzt auch nahe Null und damit so niedrig wie lange nicht mehr.

Die Kombination aus Marktzugang zu günstigen Konditionen und dem Wissen, dass die RRF-Finanzierung in einigen Jahren ankommen wird, ermöglicht es den Mitgliedstaaten, ohne große Probleme Ausgaben auf heute zu verlagern, und dabei implizit auf eine Finanzierung durch die EU-Mittel zu rechnen.

Da der Marktzugang für alle gewährleistet ist, besteht auch kaum Bedarf, die Zahlungen zu beschleunigen. Aus politischer Sicht wäre es wünschenswert zu zeigen, dass EU-Mittel schnell ankommen können. Aus wirtschaftlicher Sicht spielt es jedoch keine so große Rolle, ob die EU-Mittel ein Jahr früher oder später ausgezahlt werden. Diese „wirtschaftliche" Sichtweise beruht auf der Annahme, dass die nationalen Ausgaben dann in der Zukunft gesenkt werden können (wenn die NGEU (NextGenerationEU)-Mittel ausgezahlt werden). Wenn dies nicht der Fall ist, besteht die Gefahr, dass das NGEU-Paket de facto prozyklisch wirkt.

## Geld ist branchenübergreifend fungibel

Die von der RRF bereitgestellten Mittel werden für Projekte zur Verfügung gestellt, die von der Kommission geprüft werden und den übergeordneten Zielen der RRF entsprechen sollten, eine nachhaltige und digitale Entwicklung zu fördern. Auch hier findet man einen Kontrast zwischen der politischen und der ökonomischen Logik. Die Mitgliedstaaten werden wahrscheinlich ihre besten Projekte zur Finanzierung im Rahmen der RRF vorlegen, und zwar aus dem einfachen Grund, dass dadurch sichergestellt wird, dass sie sich für die Mittel qualifizieren können, und zwar schneller, als wenn sie Projekte von geringer Qualität vorlegen würden. Sobald die Finanzierung dieser hochwertigen Projekte durch die RRF sichergestellt ist, können die Mitgliedstaaten den freigewordenen haushaltspolitischen Spielraum nach ihren eigenen Prioritäten nutzen.

Diese Fungibilität der internationalen finanziellen Unterstützung ist nicht spezifisch für die RRF oder das NGEU-Projekt. Es gilt allgemein für alle Finanzhilfen. Eine anerkannte Maxime im Rahmen der Entwicklungshilfe ist: „Die Hilfe zahlt nicht für den Posten, für den sie bilanziert wird, sondern für die neuen Ausgaben, die sie ermöglicht."

Diese Maxime gilt auch für die RRF. Fungibilität bedeutet nicht, dass die RRF wertlos ist, aber sie impliziert, dass ihr gesamter makroökonomischer Nutzen nicht an der Qualität der Projekte gemessen werden kann, für die die Mittel ausgegeben werden. Vielmehr kommt es auf den zusätzlichen fiskalischen Spielraum an, den sie für hoch verschuldete Mitgliedstaaten schafft, und auf die Reformen, die aufgrund der allgemeinen Konditionalität der RRF durchgeführt werden. Es ist dieses letztgenannte Element, das für den Erfolg der RRF entscheidend sein wird. Auch diese Einsicht ist nicht spezifisch für die RRF- oder EU-Programme. Wie Dollar und Pritchett (1998) es ausdrücken: „Die Hilfe finanziert den gesamten öffentlichen Sektor, und die allgemeine Qualität der Politiken und Institutionen ist der Schlüssel, um eine hohe Rendite aus diesen Finanzmitteln zu erzielen."

Die Bedeutung von Strukturreformen, um Transfers produktiv zu gestalten, wird grundsätzlich von allen europäischen Institutionen und Mitgliedstaaten anerkannt. Es bleibt aber abzuwarten, ob die Kommission den Mut finden wird, einzelne nationale Reformprogramme abzulehnen. Diese Programme enthalten immer eine lange Liste guter Absichten, was es sehr schwierig macht, zu beurteilen, welche Reformen tatsächlich durchgeführt werden.

## Wie begrünt man die RRF?

Im Rahmen der RRF werden die Mitgliedstaaten ihre eigenen Projekte und Pläne zur Verwendung der Zuschüsse und Darlehen aus der RRF vorschlagen. Grundsätzlich sollten mindestens 30 % auf klimarelevante Maßnahmen entfallen. Aber wie kann man aus der RRF-Finanzierung einen „Mehrwert“, hoffentlich einen „europäischen Mehrwert“ generieren? Auch hier muss man zwischen Schein und ökonomischer Logik unterscheiden.

Die bestehenden Klimaziele der EU basieren weitgehend auf numerischen Zielen. Das prominenteste Beispiel dafür ist das Emissionshandelssystem (ETS) mit seiner Emissionsobergrenze, die über einen $CO_2$-Preis durchgesetzt wird. Das ETS deckelt die europaweiten Emissionen in der Industrie und der Energiewirtschaft. Der ETS-Preis sorgt dann für den Ausgleich zwischen Angebot und Nachfrage. Mit der bestehenden Emissionsobergrenze wird bereits sichergestellt, dass die Emission der Sektoren, die dem ETS unterliegen, bis 2030 um 55 % (gegenüber 1990) sinken werden. Deshalb bedarf es keiner Unterstützung dieser Sektoren im Rahmen der RRF. Mittel der RRF für Einsparungen von Emission in der Industrie oder Photovoltaikanlagen haben keinen Einfluss auf die Emissionen im Jahr 2030, denn diese sind schon durch das ETS gedeckelt. Subventionen für erneuerbare Energien und Industrie haben vor allem den Effekt, die Preise für Emissionszertifikate zu senken, weil der Rest der Wirtschaft weniger Anstrengungen unternehmen muss.

In anderen Branchen (Versorgernetzwerke, Fahrzeugbau, energieeffiziente Gebäude etc.) gibt es bereits eine Vielzahl nationaler und europäischer Ziele. Hier werden Projekte, die durch die RRF finanziert werden, auch die Regierungen und/oder den Privatsektor nur von ihren Verpflichtungen entbinden, diese Ziele zu erreichen. Die gleiche Logik gilt für Teile der grünen Agenda, wie die Kreislaufwirtschaft. Die allgemeine Schlussfolgerung ist, dass die Milliarden an grünen Ausgaben im Rahmen der RRF zu einem Ergebnis (in Bezug auf Emissionen oder Recycling) führen, das nicht besser ist als bereits vor der Corona-Krise geplant, es sei denn, grüne Ziele (Emissionen, Normen für Automobile, Recycling etc.) werden gleichzeitig verschärft.

Das Prinzip, dass Fonds fungibel sind, gilt natürlich nur, wenn die Märkte funktionieren. Das bedeutet, dass es Bereiche gibt, in denen die RRF einen europäischen Mehrwert erbringen kann. Ein wichtiger Bereich, in dem Märkte nur unvollkommen funktionieren, sind Netze, z. B. Stromübertragung oder Telekommunikation. Gezielte Investitionen in stärkere Netze, insbesondere Maßnahmen zur Stärkung der Verbindung zwischen den nationalen Netzen, könnten zu einer hohen europäischen Wertschöpfung führen. Was wir im Bereich der Netze brauchen, sind nicht nur die Finanzierung, sondern auch unterschiedliche Strukturen. Die Strom- und Telekommunikationsnetze werden nach wie vor von nationalen Behörden überwacht, die dazu neigen, ihre inländischen Betreiber zu schützen. Die Verbreitung erneuerbarer Energiequellen in ganz Europa würde im Prinzip große Vorteile bringen, wenn es um die Absicherung durch Solar- und Windenergie geht. Dies würde jedoch ein viel größeres Maß an Integration der Stromnetze erfordern, die nach wie vor auf der Grundlage der nationalen energiepolitischen Ziele betrieben und überwacht werden. Der wichtigste Nachteil der RRF besteht darin, dass sie sich nicht auf die Bereiche konzentriert, in denen der europäische Mehrwert am größten sein könnte.

## Schlussfolgerungen

Die europäischen politischen Entscheidungsträger:innen versuchen heute, die Fehler zu vermeiden, die in der letzten Krisenzeit gemacht wurden. Das ist bisher der richtige Ansatz gewesen. Die Maßnahmen der EZB haben entscheidend dazu beigetragen, eine weitere „Eurokrise“ zu verhindern. Das umfangreiche Paket von EU-finanzierten Zuschüssen für Mitgliedstaaten in Schwierigkeiten, die im Juli 2020 im Rahmen des NGEU-Pakets genehmigt wurden, stellt einen wichtigen Schritt auf dem Weg zur europäischen Solidarität und zur Mobilisierung gemeinsamer Steuermittel dar.

Das Vorgehen beruht auf einer Lehre, die heute vielleicht nicht anwendbar ist. Diese bezieht sich darauf, dass die Regierung einspringen muss, wenn die private Nachfrage zu schwach ist. Diese allgemeine Regel erscheint in einer „normalen“ Rezession angemessen, wenn die Nachfrage aufgrund einer Störung der Finanzmärkte oder eines anderen makroökonomischen Schocks schwächelt. Die COVID-19-Rezession ist anders, weil sie auf sehr sektorspezifischen Nachfrage- und Angebotsschocks beruht. Der wichtigste Unterschied ist die sektorale Spezifität, nicht ob es Nachfrage oder Angebot betrifft. Aggregierte Angebotsschocks sind bekanntlich „stagflationär“, d. h. sie sollten die Produktion drücken, während die Inflation steigt. Das ist heute nicht der Fall. Die aggregierten Nachfrageschocks sind deflationär, d. h. sowohl die Produktion als auch die Inflation sinken. Eine Lockerung der Fiskalpolitik ist in diesem Fall durchaus angebracht. Versuche, die Gesamtnachfrage anzukurbeln, können die Wirtschaft jedoch nicht zur Vollbeschäftigung zurückführen, wenn eine Reihe von Sektoren sowohl negativen Nachfrage- als auch Angebotsschocks ausgesetzt ist. Versuche, die gesamtwirtschaftliche Nachfrage zu stärken, dürften daher heute weniger effektiv sein, um die Erholung zu beschleunigen. Die Finanzpolitik sollte sich deshalb darauf beschränken, Einkommensverluste auszugleichen.

Die RRF stellt einen grundlegenden Fortschritt in der europäischen Integration dar. Ihr großer politischer Wert sollte jedoch nicht dazu führen, dass man einige grundlegende wirtschaftliche Prinzipien vernachlässigt. Letztlich wird der Wert der RRF nicht durch die spezifischen Projekte bestimmt werden, die sie finanziert, sondern das Maß, in dem sie Strukturreformen fördert. Eine ähnliche Feststellung gilt für den Begriff der Green Recovery. Der Nutzen für das Klima sollte nicht an den Emissionen gemessen werden, die durch die spezifischen Projekte vermieden werden, die durch die RRF finanziert werden, sondern an der Verringerung der europaweiten Gesamtemissionen, die in den nächsten zehn Jahren erreicht werden kann. Letzteres erfordert höhere $CO_2$-Preise, nicht nur eine großzügige Finanzierung für grüne Projekte über ein paar Jahre.


Literatur

Dollar, D. und L. Pritchett (1998), Assessing Aid, A World Bank Policy Research Report, http://documents1.worldbank.org/curated/en/612481468764422935/pdf/multi-page.pdf (4. Februar 2021).
